# The American Academy of Medicine and the American Medical Association

**Published:** 1896-06

**Authors:** 


### EDITORIAL.

The office of the Business Manager of THE JOURNAL is Nos. 311 and 312 Fitten Building.

The editors are not responsible for views expressed by contributors.

Articles for publication should reach this office not later than the 15th inst.

Address all communications, and make all remittances payable to THE ATLANTA MEDICAL AND SURGICAL JOURNAL, Atlanta, Ga.

Reprints of articles will be furnished, when desired, at a nominal cost. Requests for the same should always be made *on the manuscript*.

### THE AMERICAN ACADEMY OF MEDICINE AND THE AMERICAN MEDICAL ASSOCIATION.

The late meetings of these medical bodies in Atlanta were highly successful in every particular. In another place will be found a condensed report of the most important transactions of the American Medical Association.

The Academy of Medicine was the first to convene. The president, Dr. Henry M. Hurd, of the Johns Hopkins Hospital, delivered his address on "Laboratories and Hospital Work," in which he called attention to the late improvements in hospital construction, the proper equipments of a well regulated modern hospital, and recommended the addition of complete clinical, pathological, and experimental laboratories to all hospitals, in which disease in all its relations may be fully studied. Dr. J. T. Searcey, of Tuscaloosa, Alabama, read an interesting paper on "Insanity in the South," in which he stated there were some peculiar features belonging to the insane in this section of country. "In the South the whites are almost all Americans, mostly from the same English emigration. The most self-adjustable man in the world is the native American, and this is seen even when he is insane; hence the patients are more pleasant to deal with. The natural race traits of the negro show in the insane negro patients. He is passively adjustable to his environment. The negro insane can be managed with more uniformity than whites, and on the whole can be made more profitable and self-supporting." Dr. Frederick Peterson, of New York, presented a paper advocating the establishment of "Colonies for Epileptics." He said that a home should be provided for these people who are cut off from the pursuits and pleasures of life by a malady which robs them of their faculties; that a school should be established where they shall be enabled to receive an education; that they should be given an industrial education; that their cases should be studied by the best medical men and that they should be treated in a scientific manner; that there should be an abundance of land where they should be put to work in agricultural pursuits; that there should be a small hospital, a library, and other features to make the colony as homelike as possible; that most of the foodstuffs should be produced on the land; that the educational features should never be lost sight of, and in addition to the schools and industrial training, everything that can in any way be helpful in furthering such purpose should be encouraged. An interesting paper was read by Dr. J. W. Babcock, of South Carolina, on "Tuberculosis in Public Institutions." He gave some figures to show that there was an alarming amount of tuberculosis in the prisons and insane asylums of the country, and showed how it had been lessened at some institutions by a thorough cleaning of the quarters used by those who were affected. He stated that this disease spread among the inmates by the tubercle bacilli being deposited on the walls of the rooms, on the floors, and on the bedding by the sputa of the ient. After the walls had been thoroughly cleaned by scraping, and after the premises had been thoroughly disinfected, there was a marked decrease in the disease. Dr. George M. Gould, of Philadelphia, read an able and scholarly paper on "Vivisection,"

in which he paid his respects to those who were endeavoring to have vivisection prohibited by law. Far from advocating indiscriminate vivisection, he pointed out how it should be done in order to arrive at scientific conclusions. All experiments on animals should be made for a definite purpose by competent persons, and should be as nearly painless as possible. Dr. Chas. McIntire, secretary of the Academy, read a paper on "Some of the Distinguishing Characteristics of the Homo Medicus," and Dr. Elmer Lee, of Chicago, read one on "The Confusion of Pharmacy Relating to the Theory and Practice of Medicine."

In connection with the Academy of Medicine, the Association of American Medical Colleges and the National Confederation of State Boards of Medical Examiners also held their meetings. The president of the latter, Dr. W. W. Potter, delivered an address upon "Relations of Medical Examining Boards to the State, to the Schools, and to Each Other," an abstract of which will be found on another page. Mr. J. Russell Parsons, representing the Regents of the University of New York, presented an interesting paper on "Preliminary Education, Training, and Practice in New York." Methods of medical teaching were discussed in short talks by Professors Kelley of Louisville, Winslow of Baltimore, Holmes of Chicago, Vanderveer of Albany, Haggard of Nashville, Roberts of Philadelphia, and others. A banquet on Saturday night was an interesting social feature of the meeting of the Academy of Medicine, and short speeches were made by Drs. Hurd of Baltimore, Gould of Philadelphia, Potter of Buffalo, Millard of St. Paul, Bulkley of New York, Shannon of Cabaniss, Ga., Ford of Augusta, Holmes of Chicago, Gaston of Atlanta, and others.

The meeting of the American Medical Association was a representative one, both in attendance and in the character and extent of work done. The President, Dr. Cole, made a good presiding officer, and if his decisions at times were somewhat autocratic, they were based upon a desire to maintain order. His address was of broad scope and thoughtful construction, and while portions of it may be subject to some criticism, it doubtless reflects in a large measure prevailing sentiments of the conservative profession.

We have given good abstracts of the addresses on Medicine, Surgery, and State Medicine, to which the reader is referred. The Centennial Celebration of the Discovery of Vaccination was carried out by the reading of three addresses, as stated in our Society Reports, and a resolution was adopted providing for their publication in book form.

Resolutions were adopted to provide a permanent home for the Journal of the Association, and members are asked to send their ballots to the Journal during June and July. It is proposed to establish a building fund in order to provide for a proper home for the Association Journal. A resolution was also adopted protesting against the passage of a bill now before the Senate, entitled "A Bill for the Further Prevention of Cruelty to Animals in the District of Columbia," which is in reality a bill to prevent vivisection in the District.

The Report of the Treasurer showed a balance on hand of $10,-084.98.

Dr. Jerome Cochran, of Alabama, reported progress on the part of the Committee on a National Department of Public Health. The committee was enlarged so as to include a member from each State, and continued.

The annual fight on the Secretary was renewed at this meeting. The Association seems to occupy a peculiar and unfortunate position in relation to its Permanent Secretary. If it be true that the Secretary of the American Medical Association holds his position for life, and that, as was said on the floor at the late meeting, the Association cannot change its Secretary, the situation is certainly anomalous and undesirable. It is equally unfortunate for the Association to witness every year efforts to displace the present Secretary on the ground of inefficiency, and to elect somebody else to the office who could discharge the duties of the same with more competency and satisfaction to the Association. Without an unkind feeling to Dr. Atkinson, who has certainly labored long and doubtless to the best of his ability for the Association, it does seem to us that thirty years in this office ought to have satisfied him, and that the time has come for a more active and up-to-date secretary. Doubtless the opposition to Dr. Atkinson will be in evidence again at the next meeting. In the meantime, confidentially, the true and final solution of the difficult situation could be peaceably effected by a timely resignation.

Among the social features provided for the entertainment of visiting physicians and their families should be mentioned the Complimentary Concert by the young ladies of the Cox Female College, the Georgia Barbecue at Lithia Springs, and the receptions at the houses of Drs. J. S. Todd, R. B. Ridley, and A. W. Calhoun, all of which were duly patronized and enjoyed.

We close this already long article with some comments on the meeting taken from the late medical press.

The *Philadelphia Polyclinic*: The Atlanta meeting of the American Medical Association passed most enjoyably and busily. So crowded with good section work are all of these meetings now that those attending one section faithfully had little opportunity to follow the general course of events. The hospitalities extended were most kind and typical; the open clubs, the Georgia barbecue, and the beautiful receptions at the houses of eminent practitioners along the famous Peachtree street, most acceptable in themselves and having the great though negative merit of interfering very little with the scientific work. The standard was well maintained in the meeting.

The *Medical News* (New York): The recent meeting in Atlanta met the expectations of those who attended it as fully as could be expected, considering the location, in a Southern city, at this season of the year. The number in attendance, although not quite up to the average, was sufficient to afford an inspiring audience, not only in the general sessions, but also in the various sections. . . . In regard to the scientific work, the exercises were made memorable by the addresses of Osler in general medicine, and Senn in surgery. . . . The social functions fully realized the hospitable greeting of the welcoming address. All the details of the barbecue were carried out with so much system that it lacked entirely the tiresome features so often a part of such a performance. The lovely day, the delightful grove, the attractive tables reaching out their long length in the shade of the trees, all combined to make a picture that will not soon be forgotten by him who had the pleasure of enjoying it.

The *Journal of the American Medical Association:* There have been few meetings in the history of the Association which have passed off more harmoniously and more pleasantly in all respects than that just concluded at Atlanta. . . . Socially, the Southern hospitality, which is always conspicuous at these gatherings, made itself felt in such a way as to make every delegate feel at home. . . . The Georgia barbecue and the receptions at the houses of Drs. Calhoun, Ridley, and Todd are occasions long to be remembered. Indeed, if there is any criticism, it is that the Committee of Arrangements and citizens did too much to make the visit of the members agreeable and pleasant.

And so on with many others of like nature. Only one writer, so far as we have seen, or heard of, has been guilty of the inexcusable breach of good manners, accepting one's hospitality and then abusing it. This is one bilious Dr. Frederick T. Rogers, of Providence, Rhode Island, through the *Atlantic Medical Weekly*, of the same place. We regret very much that this gentleman did not enjoy his visit. He seems to be "sorry that he came," and we hasten to assure him that a similar feeling is cordially entertained at this end.

---

AN inquiry in a late *Medical Record* entitled "The American Medical Association—What is it?" has received a just and well deserved rebuke from several medical journals. The purported inquiry does not appear to have proceeded so much from ignorance as from a desire to be funny. It is said to be a custom with "Me Lord," the editor of the great medical muck-a-muck, whenever he wishes to relieve his congested gray matter of any particularly happy thought, he publishes an inquiry along the desired lines from an alleged correspondent in some remote quarter, and then proceeds to answer it. It is suggested that the call for information in the present instance is of this character. However that may be, the real author of the sentiments in question should be ashamed of them, whoever and whereever he may be. We may say for the benefit of the gentleman that the American Medical Association is a very healthy association of good doctors representing the bone and muscle of the great democratic mass of American physicians and surgeons, who think it not beneath them to keep themselves informed as to the history of our greatest national association, the parent of them all; who attend its meetings as often as they can, and seek as far as they can to build it up rather than pull it down; and that it has numbered and still numbers in its active membership a host of good men whose names will remain an honor to American medicine many years after the nameless "correspondent" of the *Record* shall have returned to nothingness and been forgotten

